# An Optimization Model for the Selection of Bus-Only Lanes in a City

**DOI:** 10.1371/journal.pone.0133951

**Published:** 2015-07-27

**Authors:** Qun Chen

**Affiliations:** School of Traffic and Transportation Engineering, Central South University, Changsha, China; University of California Berkeley, UNITED STATES

## Abstract

The planning of urban bus-only lane networks is an important measure to improve bus service and bus priority. To determine the effective arrangement of bus-only lanes, a bi-level programming model for urban bus lane layout is developed in this study that considers accessibility and budget constraints. The goal of the upper-level model is to minimize the total travel time, and the lower-level model is a capacity-constrained traffic assignment model that describes the passenger flow assignment on bus lines, in which the priority sequence of the transfer times is reflected in the passengers’ route-choice behaviors. Using the proposed bi-level programming model, optimal bus lines are selected from a set of candidate bus lines; thus, the corresponding bus lane network on which the selected bus lines run is determined. The solution method using a genetic algorithm in the bi-level programming model is developed, and two numerical examples are investigated to demonstrate the efficacy of the proposed model.

## Introduction

With the development of economy and society and the concurrent expansion of populations and city geographies, traffic congestion has become a difficult problem to manage in cities. In recent years, sustainable transportation has become a popular research topic, and the rapid development of public transportation is an important approach because high-capacity public transportation can carry more people within the same road area and release less pollution than if private cars were used. Urban public transportation modes typically include subways, buses, and public bicycles. The enhancement of existing bus operation systems lies in the design of bus-only lanes and signal priority and in the development of clean-energy buses **[1–3].** The objective of this study is to determine the layout of bus-only lanes. Urban bus lanes are separate lanes that are identified by special traffic signs and road markings or other isolation facilities and are exclusively for bus use throughout the day or during special periods of the day. To date, many countries have built bus lanes in cities, including the BRT system of Curitiba in Brazil. The planning of bus lanes is important for bus priority and can dramatically improve the attraction of buses, increase the ratio of bus trips taken by the public and therefore reduce traffic congestion **[4]**. Some studies have investigated bus lane capacities **[5, 6]**, service levels **[7, 8]** and effect evaluations **[9–12]**. However, to improve the efficiency of bus lanes, a method or model for bus lane layout should be developed. Recently, bus lane network design has been increasingly considered during the planning of bus lanes **[13, 14]**. However, there are still differences between the models and applications because the models do not reflect the transfer choice behaviors of passengers or the accessibility of all travel demands.

The selection of bus-only lanes can be considered to be a type of network design problem (NDP) that is concerned with the modification of a transportation network configuration by adding new links or improving existing ones to maximize certain social welfare objectives (e.g., total travel time over the network) within a limited budget **[15]**. NDPs are generally formulated as a mathematical problem with equilibrium constraints (MPEC) and a determinate user-equilibrium assignment model (UE) **[16–20]** or a stochastic user-equilibrium assignment model (SUE) **[21–23]** was usually applied to describe the route choice behavior of network users. However, in bus transportation systems, the route choice behavior of network users is related to the transfer times between bus lines; thus, a different traffic assignment method must be used, and different solution schemes should be pursued. Some studies have proposed models to optimize the layout of transit routes **[24–28]** but did not consider the optimization of bus lane networks or that passengers’ route choice behaviors can be influenced by transfer times. In this study, we develop a model to optimize bus-only lane networks under certain budget constraints while considering the priority sequence of transfer times in the passengers’ route choice behaviors.

The remainder of this paper is organized as follows. Section 2 formulates the model used to optimize the layout of urban bus-only lanes. Section 3 develops a solution algorithm for the model. In Section 4, two numerical examples are used to demonstrate the efficacy of the proposed model. The final section concludes the paper.

## Model Formulation

In the design of bus-only lane networks, the urban population, land-use scale and properties, urban morphology and topographic conditions should be considered; a survey and prediction of passenger flows are also necessary. To construct an appropriate model, some assumptions and preparative work must be performed:

1. Traffic zoning based on the analyses of land-use properties and topographic conditions (i.e., a zone radius of approximately 1–2 km) should be performed, and the public transit passenger flow distribution between zones should be known.
2. Because the locations of stops are unknown before the layout of bus lane networks is completed, this paper only optimizes the lines’ arrangement and does not consider stop locations to simplify the analysis; similarly, the restriction of stop capacities is ignored.
3. Although stops are not considered, passenger transfer between bus lines occurs at stop locations; thus, it is assumed that all stops within a traffic zone are collected at one point, and the distance between the stops becomes the distance between the zones’ centroids. This assumption is necessary before lines can be planned because the stop locations are unknown, and stops are not considered until the lines’ arrangement is completed.
4. The average transfer time between bus lines can be estimated based on surveys and forecasts.
5. Based on a qualitative analysis of the available conditions and the passenger flow coverage, some candidate lines are then preliminarily designed. Using the optimization model, optimal bus lines can be selected from the set of candidate lines, and the corresponding bus lane network on which the selected bus lines run can then be determined.

### Upper-level Model

An analysis of the goals of the upper-level model is described below.

The first goal of this model is to minimize the total travel time:

$$\text{min}\;\sum_{m}\sum_{n}od\left(m,n\right)\lambda_{m}^{n}$$ (1)

The second goal of this model is to minimize the total length of the bus lanes because constructing bus-only lanes is costly, requires significant amounts of land, and affects other users of the road:

$$\text{min}\;\sum_{\forall i,j\in R}l_{ij}\eta_{ij}$$ (2)

The third goal of this model is to minimize the total operation and management costs of the bus systems using the bus lanes:

$$\text{min}\;\sum_{k}\mu_{k}\gamma_{k}$$ (3)

In formulas (1), (2) and (3), *od*(*m*, *n*) is the *n* ^th^ portion of an OD (i.e., origin-destination) pair *m* of public transit passenger flows and is described by *od*(*m*) = $\sum_{n}od\left(m,n\right)$; $\lambda_{m}^{n}$ is the travel time of *od*(*m*, *n*) along its path, including the in-vehicle time, walk time, waiting time and transfer time; *i* and *j* are the zone codes; *R* is the set of traffic zones; *η*_*ij*_ is a Boolean variable that equals 1 when there is a bus lane connecting zone *i* with zone *j* and 0 otherwise; *l*_*ij*_ is the length of the bus lane connecting zone *i* with zone *j*; *γ*_*k*_ is a Boolean variable that equals 1 when the *k* ^th^ candidate bus line is selected and 0 otherwise; and *μ*_*k*_ is the operation and management cost of the *k* ^th^ candidate bus line.

The above formulas (1), (2) and (3) denote a multi-objective program in which the optimal solution is difficult to determine. However, we can use a budget constraint to replace formulae (2) and (3):

$$\sum_{\forall i,j\in R}l_{ij}\theta_{ij}\eta_{ij}+\sum_{k}\mu_{k}\gamma_{k}\leq budget$$ (4)

where *θ*_*ij*_ is the unit price of *l*_*ij*_.

In addition, accessibility constraints can guarantee that passengers from any zone can arrive at any other zone through the bus line network:

$$\sum_{\forall i,j\in R}s_{ij}\neq \infty$$ (5)

where *s*_*ij*_ refers to the travel time of the shortest path with regard to time between zones *i* and *j*. If it is assumed that the required transfer times on any valid path between any two zones is at most twice as long, *s*_*ij*_ is the shortest travel time within two times of transfer between any two zones.

### Lower-level Model

The lower-level model describes the passenger flow assignment on the bus routes. According to Wang [29], the priority order of the path-finding is as follows: through paths without transfer → paths with one transfer → paths with two transfers → etc. Although Wang [29] introduced the improved logit path-selection model into the calculation of public transit passenger flow assignment on the paths, it is easy to understand the following concept for paths on a given priority level. (1) For travelers, particularly commuters, the required average travel time along each possible path is known before their trips because the bus routes are generally fixed. (2) Under the conditions that the number of buses and departure frequencies are fixed, the travel time of each route generally has no direct connection with the number of passengers on the route; thus, traffic impedance can be represented by a relatively constant value if passenger comfort is ignored. Additionally, for paths on a given priority level, the order of travelers’ selection should be as follows. The shortest path with regard to time or the path having the minimum traffic impedance while considering the travel time, fees and transfer times should be the passenger’s first choice. In addition, due to the limitations of route capacities, some passengers will be left at bus stops if no additional capacity is available. The capacity of one route is related to the number of seats on the bus, the departure frequency and the load factor. Capacity limitations typically make passengers unable to select crowded routes; those passengers must then select paths with a lower priority level that may require transfers.

**Fig 1:**
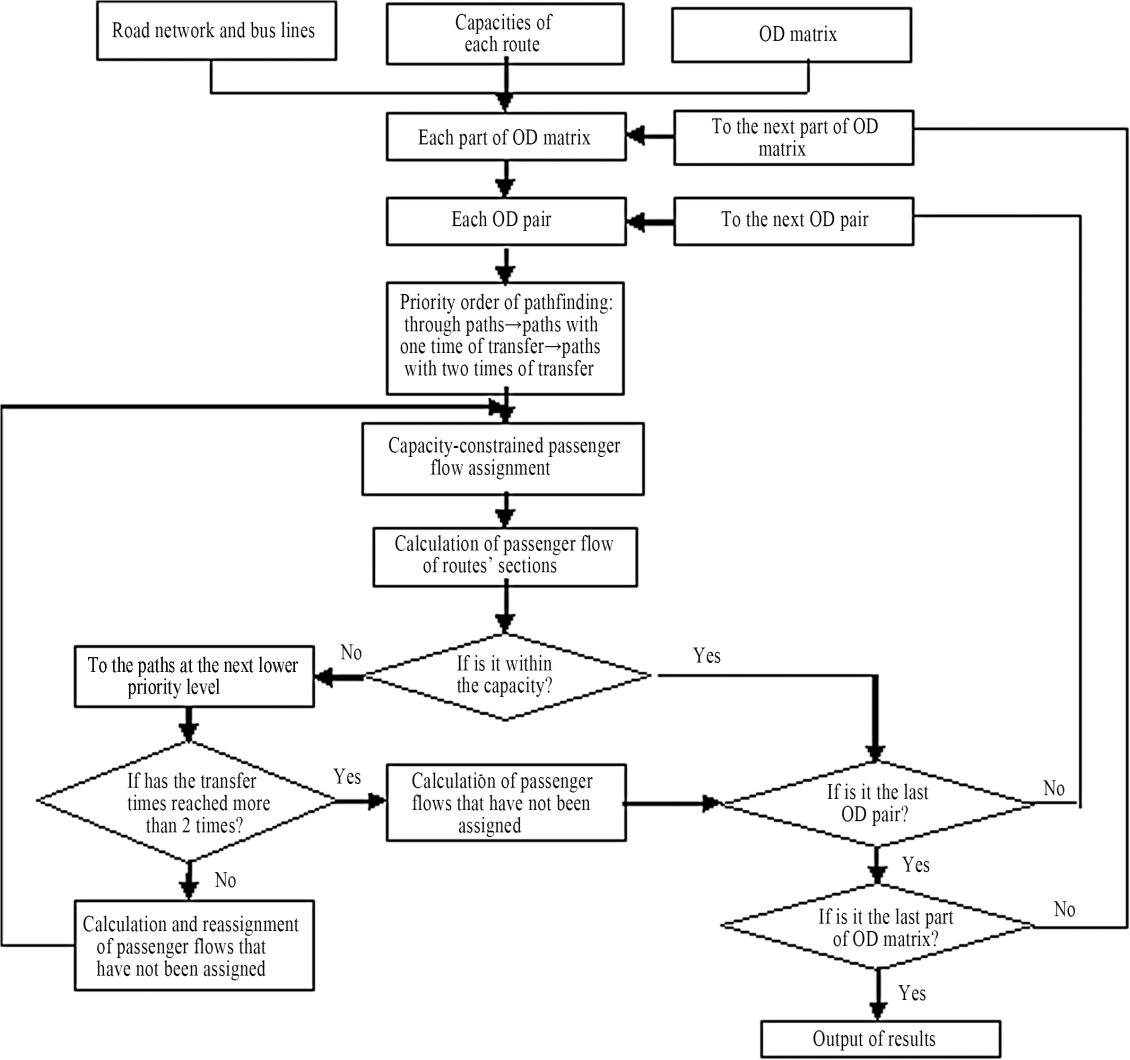
Logic flow diagram of the capacity-constrained traffic assignment.

From the above analysis, the capacity-constrained traffic assignment method can be applied. Therefore, the order of travelers’ path selection should be as follows: through paths → paths with one transfer → paths with two transfers. Additionally, for paths on the same priority level, the shortest path with regard to time is selected first; the other longer paths or paths at a lower priority level are not selected until the capacity of one route is reached. When the capacity-constrained traffic assignment model is applied, the OD matrix of public transit passenger flow should be divided into several parts, and all parts should be assigned to the network. Fig 1 shows the logic flow diagram of the capacity-constrained traffic assignment.

Analyses of the public traffic impedance and path-finding methods are introduced as follows.

(1) Public traffic impedance

The public traffic impedance is an index that comprehensively considers travel time, fees and passenger comfort. Wang [29] introduced an expression of the public traffic impedance that considers the travel time and the ticket price that vary with the distance; however, because many cities have applied single-entrance ticket systems and the fee paid for riding on buses is comparatively small compared to peoples’ income, this study primarily considers travel time and ignores fees:

$$T=D/V+(t_{\text{w}}+t_{\text{i}}/2+\omega t_{\text{h}})/60$$ (6)

where *T* is the total travel time (hour), *D* is the distance (km), *V* is the average speed of the buses (km/h), *t*_w_ is the sum of the time to walk to and from bus stops (min), *t*_i_ is the departure interval (min), *ω* is the transfer times and *t*_h_ is the time to complete a transfer (min). When passenger comfort is ignored, public traffic impedance can be relatively constant.

(2) Path-finding methods

The steps used to find paths are described as follows:

① Take bus line *L*_1_ passing through the start point *I*;
② Determine if line *L*_1_ passes through the end point *J*; if not, then return to step ①; if yes, then go to the next step;
③ Record the path from point *I* to *J* using bus line *L*_1_ *;*
④ If there are other lines passing through point *I*, then return to step ①; if not, end.

The steps used to find paths with one transfer are described as follows:

① Take bus line *L*_1_ passing through the start point *I*;
② Take bus line *L*_2_ passing through the end point *J*;
③ Determine if bus line *L*_2_ intersects bus line *L*_1_; if not, return to steps ② and then ①; if yes, then go to the next step;
④ Record the paths from *I* to *J* using bus lines *L*_1_ and *L*_2_;
⑤ If there are other lines passing through the end point *J*, then return to step ②; if there are other lines passing through the start point *I*, then return to step ①; if not, end.

The steps used to find paths with two transfers are described as follows:

① Take bus line *L*_1_ passing through the start point *I*;
② Take bus line *L*_2_ intersecting with bus line *L*_1_;
③ Take bus line *L*_3_ intersecting with bus line *L*_2_;
④ Determine if bus line *L*_3_ passes through the end point *J*; if not, then return to steps ③, then ② and then ①; if yes, then go to the next step;
⑤ Record the paths from *I* to *J* using bus lines *L*_1_, *L*_2_ and *L*_3_;
⑥ If there are other lines that intersect bus line *L*_2_, then return to step ③; if there are other lines that intersect bus line *L*_1_, then return to step ②; if there are other lines that pass through the start point *I*, then return to step ①; if not, end.

After all paths have been found, and the buses’ speed and transfer times have been calculated, the travel time can then be calculated.

## Solution Algorithm

The upper-level model is a 0–1 programming problem with constraints; thus, a genetic algorithm’s 0–1 coding is suitable for solving this problem.

Applying 0–1 coding, and assuming that there are *Y* candidate lines in total, the chromosome can be expressed as follows:

[0,1,1,0,…,0,0,1]

where the number 1 denotes that this route is selected, and the number 0 denotes that this route is not selected.

Steps for solving the model using the genetic algorithm are described as follows:

*Step* 1. Initialization. Set the population size, the chromosome length, the number of iterations, the crossover rate, and the mutation rate.
*Step* 2. Apply 0–1 coding to randomly produce the initial population, and set *gen* = 1.
*Step* 3. Pass the solutions denoted by the chromosomes of the population to the lower-level model, and find the paths for each OD pair through the capacity-constrained traffic assignments. Then, return to the upper-level model, and calculate the fitness of each individual and the excess of the constraint restrictions; for feasible solutions, the excess is 0. If *gen* = *MaxGen* (i.e., the maximum number of iterations has been reached), output the optimal individual; otherwise, go to *step* 4.
*Step* 4. Use the roulette method based on ranking to select the next generation’s population.
*Step* 5. In terms of the crossover rate *P*_*c*_, perform a single-point crossover operation.
*Step* 6. In terms of the mutation rate *P*_*m*_, perform a mutation operation, set *gen* = *gen* + 1, and then return to *step* 3.

## Numerical Examples

### Example 1

**Fig 2:**
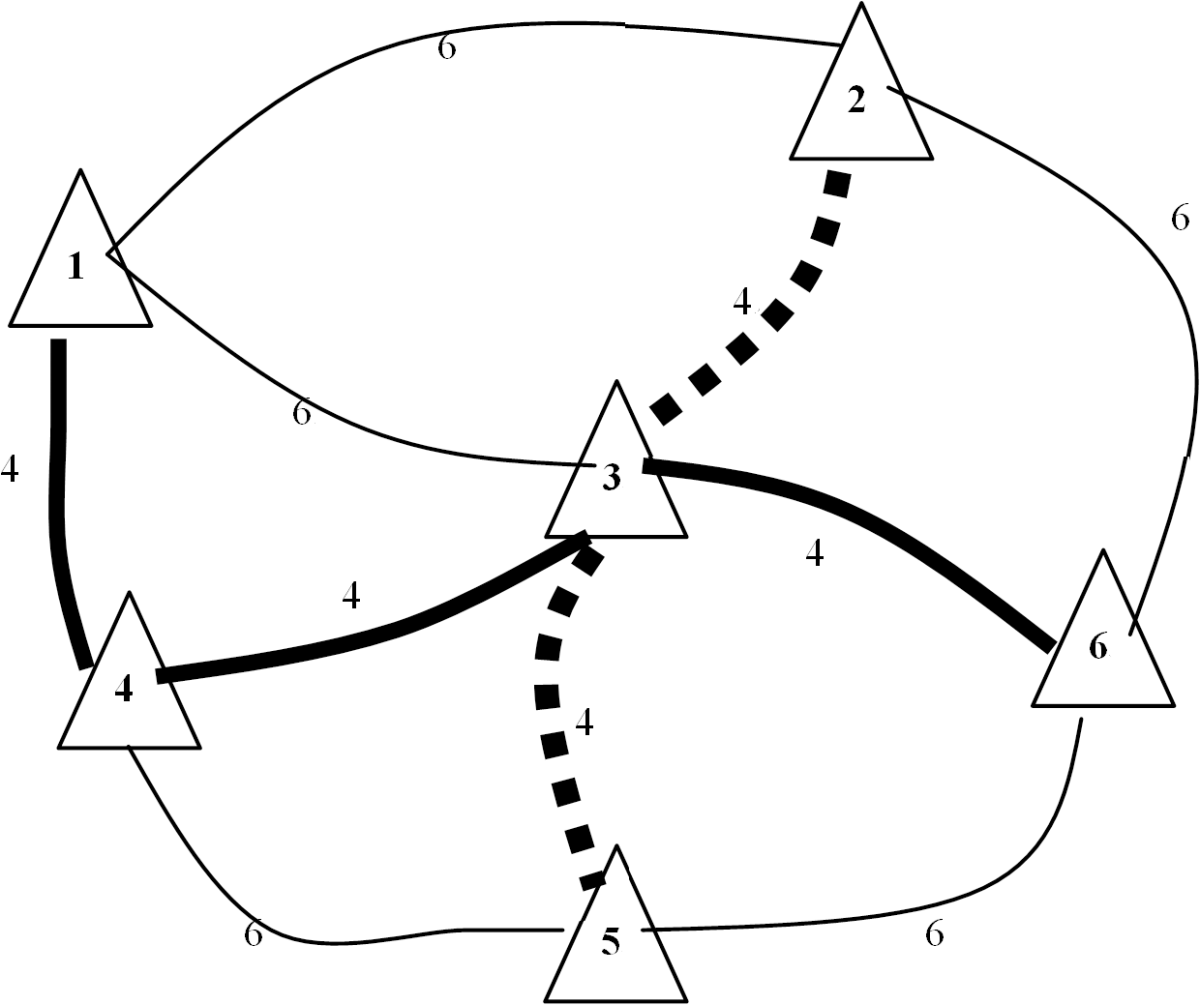
Positions of the zones in example 1.

In this example, bus lanes are built to connect 6 primary zones. Fig 2 shows the positions of the zones and the in-vehicle time of each side (unit: min). The transfer time between any two lines is set equal to 2 min, and the lengths of the sides are as follows: 1–4 is 2 km; 4–5 is 3 km; 5–6 is 3 km; 6–2 is 3 km; 1–2 is 3 km; 1–3 is 3 km; 2–3 is 2 km; 3–6 is 2 km; 3–4 is 2 km; and 3–5 is 2 km. The candidate bus lines are: ① 1–3–6; ② 1–4–3–6; ③ 4–5–6–2; ④ 1–4–5–6; and ⑤ 2–3–5.

It is assumed that the total length of the bus lines cannot exceed 10 km, and the capacity in one direction of each line is 2000 persons per day. Table 1 shows the OD pairs.

**Table 1:** OD pairs (unit: persons per day) in example 1.

D	1	2	3	4	5	6
O						
1	0	100	200	600	50	200
2	100	0	500	100	400	150
3	200	100	0	400	400	400
4	600	100	400	0	100	400
5	50	400	400	100	0	100
6	200	150	400	400	100	0

The chromosome length is equal to 5 (i.e., the number of candidate lines), the size of the population is equal to 150, the crossover rate is set equal to 0.7 and the mutation rate is set equal to 0.05. Using Matlab 7.0 software, the optimal solution can be obtained after 15 iterations, and the bus lines denoted by the optimal solution are ② 1–4–3–6 and ⑤ 2–3–5. The total travel time of the passengers is equal to 26200 min. The flow rates in one direction are shown in Table 2; two directions have the same flow rates. The transfer flow rates at node 3 between the two lines are both equal to 600 persons per day.

**Table 2:** Flow rates in one direction (unit: persons per day).

Link	1–4	4–3	3–6	2–3	3–5
Flow	1150	1550	1250	1250	1050

Thus, the bus lanes to be built include lanes 1–4, 4–3, 3–6, 2–3, and 3–5.

### Example 2

**Fig 3:**
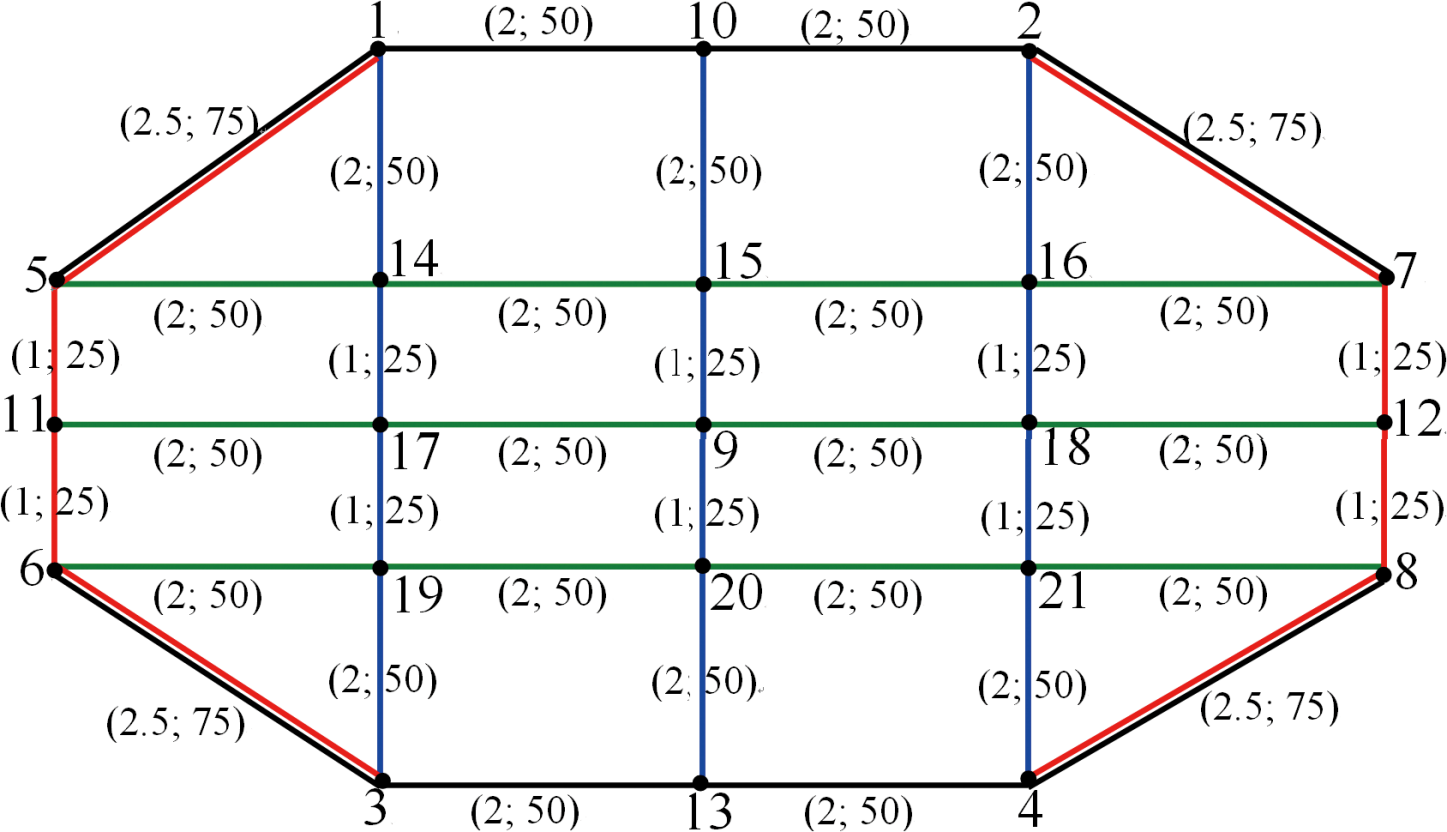
Bus line network used in example 2.

There are 10 candidate bus lines in total shown in Fig 3: bus line ① (5-1-10-2-7), whose capacity in one direction is 200 persons per hour; bus line ② (6-3-13-4-8), whose capacity in one direction is 200 persons per hour; bus line ③ (1-5-11-6-3), whose capacity in one direction is 200 persons per hour; bus line ④ (2-7-12-8-4), whose capacity in one direction is 200 persons per hour; bus line ⑤ (5-14-15-16-7), whose capacity in one direction is 400 persons per hour; bus line ⑥ (11-17-9-18-12), whose capacity in one direction is 400 persons per hour; bus line ⑦ (6-19-20-21-8), whose capacity in one direction is 400 persons per hour; bus line ⑧ (1-14-17-19-3), whose capacity in one direction is 300 persons per hour; bus line ⑨ (10-15-9-20-13), whose capacity in one direction is 300 persons per hour; and bus line ⑩ (2-16-18-21-4), whose capacity in one direction is 300 persons per hour.

The bus travel time (unit: min) and the construction cost (unit: million dollars) of each side in Fig 3 are presented in parentheses. The operation and management costs of each bus line is 100 million dollars, and the total budget for this project is set equal to 1,800 million dollars. The transfer time between any two bus lines is set equal to 5 min. The origin-destination (OD) traffic distribution is shown in Table 3.

**Table 3:** OD pairs (unit: persons per hour) in example 2.

D	1	2	3	4	5	6	7	8	9
O						
1	0	90	0	35	90	80	70	0	0
2	50	0	40	0	70	95	80	0	35
3	0	35	0	0	70	60	40	0	0
4	35	80	0	0	30	0	40	0	40
5	90	50	65	0	0	70	90	0	0
6	70	95	50	0	70	0	0	80	40
7	80	90	0	0	70	30	0	0	0
8	0	30	0	0	30	80	0	0	50
9	0	20	0	40	0	30	0	30	0

**Fig 4:**
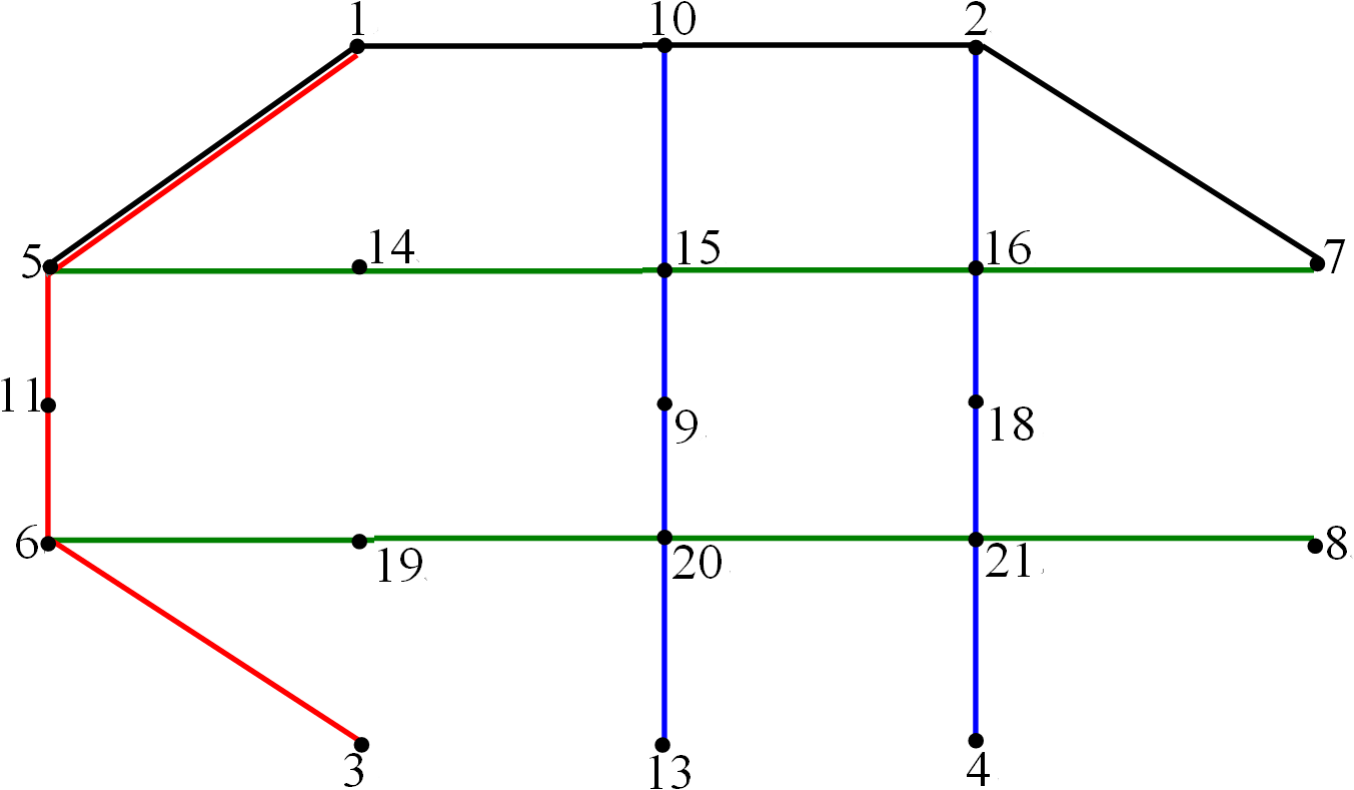
Optimized bus line network.

The length of the chromosome is equal to 10 (i.e., 10 candidate lines), the size of population is equal to 100, the crossover rate is set equal to 0.7 and the mutation rate is set equal to 0.1. The loaded ratios of the OD traffic distribution in the capacity-constrained traffic assignments are 0.2, 0.2, 0.15, 0.1, 0.1, 0.05, 0.05, 0.05, 0.05 and 0.05 in sequence. Using Matlab 7.0 software, the optimal solution can be obtained after 15 iterations, and the bus lines denoted by the optimal solution are: ①, ③, ⑤, ⑦, ⑨ and ⑩. The total travel time of passengers is equal to 26 810 min. The flows of each bus line are shown in Table 4, and the transfer flows between the two lines are shown in Table 5. The optimized bus line network is shown in Fig 4; thus, the bus lanes to be built include lanes 1–5, 1–10, 10–2, 2–7, 5–14, 14–15, 15–16, 16–7, 6–19, 19–20, 20–21, 21–8, 5–11, 11–6, 3–6, 10–15, 15–9, 9–20, 20–13, 2–16, 16–18, 18–21, and 21–4.

**Table 4:** Flow rates of each bus line.

Bus line ①: 5-1-10-2-7		Bus line ③: 1-5-11-6-3	
Link	Flow rate (persons per hour)	Link	Flow rate (persons per hour)
5→1	209	5→1	191
1→5	211	1→5	208
1→10	206	5→11	209
10→1	203	11→5	225
10→2	217	11→6	209
2→10	222	6→11	225
2→7	198	6→3	155
7→2	206	3→6	205
Bus line ⑤: 5-14-15-16-7		Bus line ⑦: 6-19-20-21-8	
Link	Flow rate (persons per hour)	Link	Flow rate (persons per hour)
5→14	319	6→19	291
14→5	330	19→6	297
14→15	319	19→20	291
15→14	330	20→19	297
15→16	231	20→21	226
16→15	251	21→20	260
16→7	201	21→8	110
7→16	145	8→21	190
Bus line ⑨: 10-15-9-20-13		Bus line ⑩: 2-16-18-21-4	
Link	Flow rate (persons per hour)	Link	Flow rate (persons per hour)
10→15	19	2→16	149
15→10	11	16→2	268
15→9	145	16→18	95
9→15	128	18→16	291
9→20	210	18→21	95
20→9	238	21→18	291
20→13	0	21→4	75
13→20	0	4→21	225

As shown in Table 4, the flow rates of some links are marginally larger than their capacities because the OD traffic distribution is proportionally loaded onto the network in the traffic assignment, which may cause in-vehicle congestion among passengers.

**Table 5:** Transfer flow rates between pairs of lines.

Node 1		Node 2		Node 5		Node 6		Node 7	
Direction	Flow rate	Direction	Flow rate	Direction	Flow rate	Direction	Flow rate	Direction	Flow rate
①→③	0	①→⑩	19	①→③	74	③→⑦	65	①→⑤	0
③→①	71	⑩→①	19	③→①	0	⑦→③	63	⑤→①	0
				①→⑤	0				
				⑤→①	47				
				③→⑤	145				
				⑤→③	75				
Node 10		Node 15		Node 16		Node 20		Node 21	
Direction	Flow rate	Direction	Flow rate	Direction	Flow rate	Direction	Flow rate	Direction	Flow rate
①→⑨	19	⑤→⑨	126	⑤→⑩	96	⑦→⑨	238	⑦→⑩	146
⑨→①	11	⑨→⑤	117	⑩→⑤	173	⑨→⑦	210	⑩→⑦	100

## Conclusions

The rapid development of public transportation can alleviate serious traffic congestion in cities, provide comfortable and convenient transportation service for more people than private vehicles and achieve sustainable development in urban transportation. Bus-only lanes can allow buses to operate in special lanes without disturbances from other forms of traffic, allowing buses to run faster and making public transportation more attractive to passengers. A bi-level optimization model used to determine the layout of bus-only lanes is proposed in this study. The goal of the upper-level model is to minimize the total travel time of passengers while considering accessibility and budget constraints; the goal of the lower-level model is to develop a capacity-constrained traffic assignment model that describes passenger flow assignments on bus lines. Using this bi-level model, optimal bus lines are selected from a set of candidate bus lines; thus, the corresponding bus lanes are identified. Two numerical examples are used to demonstrate the effectiveness and feasibility of the proposed model.

The primary contributions of this study are as follows. (1) The transfer choice behaviors of passengers are considered in the proposed model. In passengers’ route choice behaviors, a priority sequence of transfer times is considered first, and fewer transfer times are considered to be preferred. Then, for paths with the same transfer times, the path with the shortest time is selected, and the other longer paths or paths with more transfer times are not selected until the capacity of one route is reached. In addition, path-finding methods including finding through paths, paths including one transfer and paths including two transfers are analyzed. (2) A bi-level model is proposed to optimize the layout of bus-only lanes within a given area; the upper-level model evaluates each scheme of the layout of bus-only lanes, and the lower-level model calculates the passenger flow assignments on each bus route under the scheme provided by the upper-level model. The results of the traffic assignments are then returned to the upper-level model to evaluate each scheme of the layout of bus-only lanes. (3) The accessibility of all travel demands can be guaranteed through accessibility constraints in the proposed model, which ensure that passengers from any zone can arrive at any other zones using the bus line network.

However, some issues should be addressed in the future. For example, this study did not consider bus stop capacities, passenger comfort or riding fees. These issues must be further investigated in the future.
